# Cellulose Acetate and Polycaprolactone Fibre Coatings on Medical-Grade Metal Substrates for Controlled Drug Release

**DOI:** 10.3390/polym16142006

**Published:** 2024-07-13

**Authors:** Catarina Cidade do Carmo, Miguel Brito, J. P. Oliveira, Ana Marques, Isabel Ferreira, Ana Catarina Baptista

**Affiliations:** 1 CENIMAT|i3N, Department of Materials Science, NOVA School of Science and Technology, NOVA University Lisbon, 2829-516 Caparica, Portugal; cp.carmo@campus.fct.unl.pt (C.C.d.C.); m.brito@campus.fct.unl.pt (M.B.); jp.oliveira@fct.unl.pt (J.P.O.); acl.marques@fct.unl.pt (A.M.); 2 Physics Department, Faculty of Sciences, University of Lisbon, 1749-016 Lisbon, Portugal

**Keywords:** cellulose acetate, polycaprolactone, ibuprofen, electrospinning, blow spinning, drug release

## Abstract

This study explores a method that has the potential to be cost effective in inhibiting biofilm formation on metallic prostheses, thereby preventing rejection or the requirement for replacement. A cost-effective metal alloy used in biomedical implants was chosen as the substrate, and ibuprofen (Ibu), a well-known anti-inflammatory drug, was selected for drug release tests for its widespread availability and accessibility. Multilayer coatings consisting of cellulose acetate (CA), polycaprolactone (PCL), and chitosan (CHI), with or without ibuprofen (Ibu) content, were applied onto medical-grade stainless steel (SS-316 type) through electrospinning, electrospray, or blow spinning. The adhesion of the CA, PCL, and layered CA/PCL membranes, with thicknesses ranging from 20 to 100 μm, to SS substrates varied between 0.15 N and 0.22 N without CHI, which increased to 0.21 and 0.74 N, respectively, when a CHI interlayer was introduced by electrospraying between the SS and the coatings. Although drug release in a simulated body fluid (SBF) medium is predominantly governed by diffusion-driven mechanisms in all single- and multilayer coatings, a delayed release was noted in CA coatings containing Ibu when overlaid with a PCL coating produced by blow spinning. This suggests avenues for further investigations into combinations of multilayer coatings, both with and without drug-imbued layers.

## 1. Introduction

Metals, due to their high strength, fracture resistance, good formability, and, most importantly, biocompatibility, which prevents corrosion of the implant, are the most ancient and commonly used materials in implants. Stainless steel (SS), titanium and its alloys, cobalt-based alloys, and tantalum are extensively used in implantable devices [1]. SS, particularly the 316 and 316 L types, is favoured for orthopaedic surgeries like fracture fixation and bone plates, owing to its high shear strength compared to titanium alloys and the ability to vary corrosion resistance based on the alloy. Its versatility, cost-effectiveness, and ease of handling also contribute to its popularity [1]. However, SS implants can lead to complications such as allergic reactions to wear debris, corrosion-induced toxicity, and device degradation.

Biofilm development causes recurrent or persistent infections due to tissue damage, dysfunction, and the consequent necessity to replace implants, which accounts for about 15% of all orthopaedic operations [2,3]. Preventing biofilm formation on implant materials in contact with surrounding tissues and physiological fluids is then crucial.

Surface modifications, treatments based on nanoparticles, biocompatible polymer coatings, and adjustments in antibiotic concentration are among the principal strategies for preventing and tackling biofilms [2]. Employing blends of polymers as coating materials for controlled drug delivery systems can offer substantial benefits, including the capacity to tailor drug release patterns, mechanical attributes, and release mechanisms, alongside film formation and storage stability [2].

Cellulose acetate (CA), a cellulose derivative, is a plentiful, natural, biodegradable, biocompatible, non-toxic polymer that can form membranes of nanofibres with high chemical and mechanical stability, excellent water affinity, and high porosity and thus serve as an effective drug carrier [4,5]. Polycaprolactone (PCL), a synthetic polymer that is both biocompatible and biodegradable, degrades in physiological conditions through the hydrolysis of its ester bonds, making it suitable as an implantable biomaterial [6]. PCL membranes form a randomised fibrous and porous matrix enhancing mechanical strength and allowing for cell proliferation and fluid absorption. PCL has been modified with biopolymers like CA to improve hydrophilicity, strength, and biocompatibility [7].

Chitosan (CHI) is a naturally occurring cationic and highly basic linear polysaccharide derived from the N-deacetylation of chitin [8]. It boasts antimicrobial properties that promote cell proliferation, is biodegradable and biocompatible, and provides excellent mechanical resistance to biomaterials based on it [9]. CHI can be formed into films, fibres, beads, powders, and solutions. The cationic nature of CHI attracts negatively charged cytokines and growth factors, aiding in protection and concentration, thereby hastening healing [10]. Ibuprofen (Ibu), in this study, was used solely to confirm drug retention and release from the CA and PCL fibres. The choice was due to its availability and low cost compared to antibacterial drugs.

Electrospinning/electrospray (ES) and blow-spinning (BS) techniques were chosen for membrane fabrication due to their versatility, simplicity, and scalability [11]. BS is a new method for producing polymeric micro- and nanofibres, emerging as a fast, safe, low-cost, and attractive alternative to ES. Unlike ES, BS does not require a voltage source to establish an electric field, making it more energy efficient. Additionally, polymers do not need to be conductive for the BS process to work effectively. Despite its numerous advantages, BS may produce less uniform fibre diameters than ES. This variation in fibre uniformity is contingent upon factors such as the polymer solution concentration, which directly affects solution viscosity [12]. Previous studies have used Ibu in CA fibres for bandages and wound dressings to reduce inflammation, with fibres dissolving rapidly to release Ibu. This suggests the potential for using fast-dissolving drug delivery for poorly water-soluble drugs like Ibu [13].

Other research shows that CHI coatings can offer short-term corrosion protection due to their rapid resorption rate, while their superior biocompatibility and bioactivity bring additional biological performance benefits. PCL coatings improved mechanical adhesion to AZ91 alloy substrates and enhanced osteoblastic cell spreading and elongation compared to untreated substrates [14]. The combining of CHI and PCL in a single coating system offers unique properties such as antimicrobial ability, osteoinductivity, and strong surface adhesion [14]. Previously, we investigated dip-coating and ES techniques to prepare alternating layers of CHI and CA fibres containing antibiotics as protective coatings and drug delivery vehicles for metal implants [15,16]. This work builds upon those advancements, by studying the impact of multilayer membranes composed of CA and PCL on the drug release mechanism. Furthermore, it was determined that CHI layers improve the adhesion of films to SS substrates.

## 2. Materials and Methods

### 2.1. Materials

The CA (Sigma-Aldrich, St. Louis, MO, USA, average Mn ~50,000 by GPC, 39.7 wt.% acetyl) solution, 12% *w*/*v*, was prepared using dimethylacetamide (≥99.8%, Carlo Erba, Val-de-Reuil, France) and acetone (≥99.5%, Honeywell, Raunheim, Germany) in a 1:2 ratio. The PCL (Sigma-Aldrich, Poole, UK, average Mn ~80,000) solution, 5% *w*/*v*, was produced from a 7:3 ratio of dichloromethane (Carlo Erba, Val-de-Reuil, France) and dimethylformamide (≥99.9%, Carlo Erba, Val-de-Reuil, France). Ibu (Farma-Quimica Sur, Málaga, Spain), 1 mg, was incorporated into both the CA and PCL solutions at a concentration of 0.5 g/L. The CHI (Sigma-Aldrich, St. Louis, MO, USA) solution, 0.5% *w*/*v*, was made using a 1:1 mixture of ultrapure water and ethanol (≥99.8%, Honeywell, Germany) and acetic acid (≥99.7%, Sigma-Aldrich, St. Louis, MO, USA) at 1% *v*/*v*.

### 2.2. Methods

#### 2.2.1. SS Substrate Preparation

Medical-grade SS substrates (GoodFellow Cambridge Ltd., Huntingdon, UK, 316 L), simulating the surface of prosthetics, were utilised. The membranes were then replicated onto the SS substrates, which had been cleaned by prior mechanical and chemical abrasion to remove surface oxides and create anchoring sites for improving film/membrane adhesion, as previously outlined [15]. Figure S1 in the Supporting Information shows the optical microscopy images of the SS substrate before and after treatment.

#### 2.2.2. Membrane Preparation

The CA solution was utilised to fabricate membranes via electrospinning with a flow rate of 0.2 mL/h and an applied voltage of 18 or 20 kV, for membranes with and without Ibu, respectively. The PCL membranes were produced using either electrospinning or blow-spinning techniques. For electrospinning, the flow rate was 0.5 mL/h and the voltage was 18 kV. CHI was applied onto SS substrates immediately prior to the CA or PCL layers using electrospray at 20 kV and a flow rate of 0.2 mL/h. Common parameters in all the membranes’ productions include a 21G needle, a needle–target distance of 15 cm, humidity between 30 and 50%, and temperature range of 20–28 °C. The blow-spinning process involved extruding polymer solution through an inner nozzle with high-pressure gas, creating a jet from the tip of a Taylor cone, which then propagated towards the collector. The CA and PCL membranes were initially deposited on aluminium foil, left for 24 h to dry, and then removed with a scalpel. To ensure that a consistent amount of Ibu was incorporated into all sample proportions, we utilised the entire Ibu solution (containing 1 mg) in the production of the CA and PCL membranes. This approach was designed to maximise the loading of Ibu into the membranes.

The experimental setup of the electrospinning and electrospray and the way the membranes were deposited on SS substrates are shown in Figure S2 in the Supporting Information. The SS substrates were attached with aluminium adhesive tape to an aluminium-foil-coated target; the setup included a copper ring attached to the syringe needle to collimate the flow and to reduce fibre dispersion.

The macroscopic and microscopic images of the obtained CA membrane are shown in Figure S3 in the Supporting Information. Figure S4 in the Supporting Information shows the schematics of the studied samples: single and multilayers with and without Ibu deposited in SS substrate.

#### 2.2.3. Morphological and Chemical Characterisation

The surface morphology of the substrates and coatings was examined using a scanning electron microscope (SEM Hitachi S2400) with a 20 kV energy beam. Carbon tape was used to fix the samples in the sample holder. Raman spectroscopy, utilising a Alpha 300 RAS microscope (WITec, Ulm, Germany) equipped with a 532 nm wavelength laser (with power set to 1.5 mW), was performed to analyse the composition of both ES-produced membranes of CA and PCL. This analysis was carried out both prior to Ibu loading and following its release to ensure the process was complete. The resulting spectra were compared with those of the raw CA, PCL, and Ibu materials, previously measured and reported in our prior work [16,17,18]. The main peaks detected are summarised in Table S1 of the Supporting Information.

#### 2.2.4. Drug Release Methodology

A simulated body fluid (SBF) solution was prepared following a standard protocol [19]. To conduct the drug release study, an absorption–concentration calibration curve was first performed using the following methodology. A solution of SBF–Ibu at a concentration of 20 mg/L was prepared; the SBF–Ibu solution was then diluted sequentially, and the absorbance spectra for each concentration were obtained using UV–VIS spectrophotometry (T90+ UV/VIS Spectrometer, PG Instruments Ltd., Leicester, UK).

The drug release from the membranes was studied by immersing each one in 20 mL of SBF at 37 °C and regularly measuring the absorption spectrum for comparison with a calibration curve (absorption at λ = 222 nm vs. drug concentration in SBF). A similar absorption–concentration calibration curve for Millipore water–Ibu was established in a prior study [18] using the same method. Figure S5 in the Supporting Information shows stainless-steel substrates coated before and after release.

The calibration curves of the Ibu in SBF are shown in Figure S6 in the Supporting Information (similar curves were obtained for water medium). The drug release calculations and models used are also presented in the Supporting Information.

To have an idea how membranes behave in the SBF solution, swelling tests were performed by weighing the membranes and immersing them in SBF solution at 37 °C, to simulate body temperature. After immersion and prior weighing, the membranes were dried with absorbent paper. This process was initially repeated 6 times for periods of 20 min and, then, at 24 h intervals for 5 days. The swelling ratio was calculated according to the equation: Swelling Ratio (%) = (W_S_/W_0_) × 100, where W_0_ is the original weight of each sample before swelling and Ws is the dry weight after the swelling process.

Contact angle measurements were conducted at 23 °C using an OCA20 contact angle measuring equipment from DataPhysics Instruments. Water drops, with a volume of approximately 5 μL, were placed on the sample surface and the contact angle was measured after 1 s of the drop deposition. The image acquisition, analysis, and contact angle determination were carried out with the SCA20 software v.4.3.1.7 (Dataphysics Instruments GmbH, Filderstadt, Germany).

#### 2.2.5. Mechanical Tests

The stress testing was done by cutting 1 × 1.5 cm^2^ membranes, measuring their thickness, and fixing them in a universal mechanical testing machine (Shimadzu AG-50kNG with the “Trapezium2” software version 2.2). The membranes were stretched at 2 mm/minute by the 1 cm side of the samples, with increasing force up to 10 N, until the membrane tore. Figure S7 in the Supporting Information shows an example of a tensile test and its obtained curve.

The peeling-off test enhances the adhesion properties of the polymeric film bonded to the SS substrate fixed in a universal mechanical testing machine (Shimadzu AG-50kNG with the “Trapezium2” software). The film was covered with adhesive tape. The SS tip was attached to one of the claws, and the adhesive tape tip was attached to the moving claw, which was pulled at 5 mm/min, with an increasing force up to 5 N, until it detached from the substrate. This procedure was carried out in films made with and without CHI, to evaluate its influence on adhesion. Figure S8 in the Supporting Information shows the schematics of the peeling-off tests and an example of the obtained curve.

## 3. Results and Discussion

**Figure 1:**
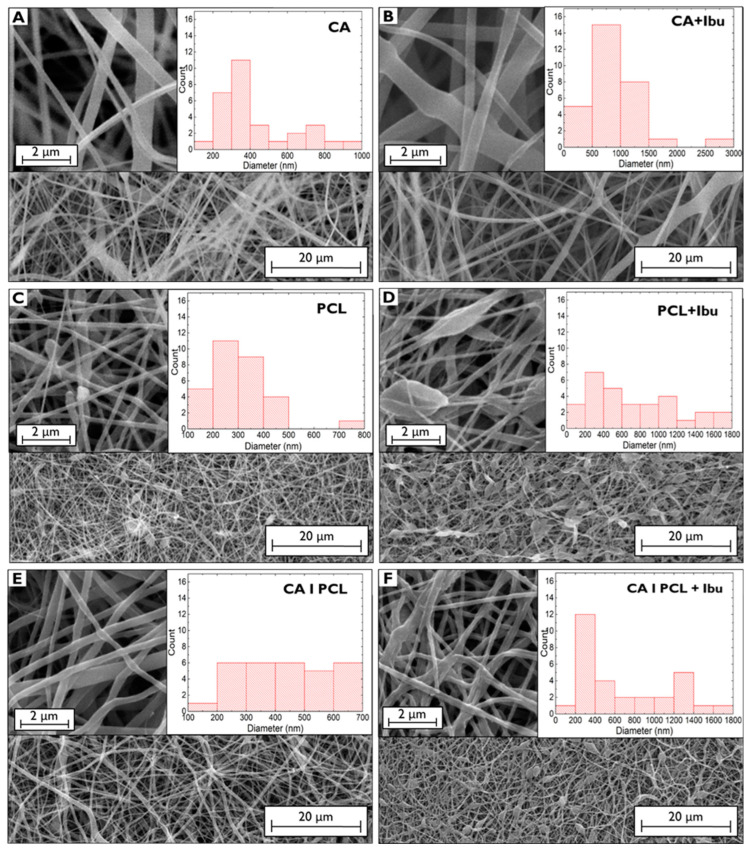
SEM images and respective distribution of diameters of CA without (**A**) and with (**B**) Ibu, PCL without (**C**) and with (**D**) Ibu, and CAǀPCL membranes without (**E**) and with (**F**) Ibu.

The SEM images of the CA, PCL, and layered CA|PCL membranes are shown in Figure 1. One can observe the morphology and a histogram depicting the average diameters from five measurements taken using the higher-magnification SEM image and ImageJ^®^ 1.53t software (NIST, Gaithersburg, MD, USA).

The displayed images show that the incorporation of Ibu into the fibres led to an overall enlargement of the fibres. For instance, CA membranes without Ibu had predominantly diameters in the range of 300–400 nm, whereas with Ibu, it ranged 600–1000 nm. The increased solution viscosity due to Ibu’s addition might lead to this enlargement in the diameter of fibres, compared to those of CA electrospun without Ibu [20,21]. This trend was similarly observed in PCL membranes without Ibu, diameters in the range of 200–700 nm and a high number of fibres with broader diameters, up to 1800 nm, with Ibu. This behaviour and the altered morphology have been also attributed to the increase in solution viscosity when Ibu is added [22]. However, it is unclear whether the origin of broader zones in the PCL fibres is related to the accumulation of Ibu or whether the increased viscosity of the PCL and Ibu solution provokes these entanglements and/or fibre fusion. The SEM image of CA|PCL is indeed the top view of the PCL membrane since this membrane is deposited on top of a CA membrane. Although the same process and conditions are applied, the difference in the range of diameters without and with Ibu, reflects also the possible fusion of PCL with the CA underlayer during deposition.

**Figure 2:**
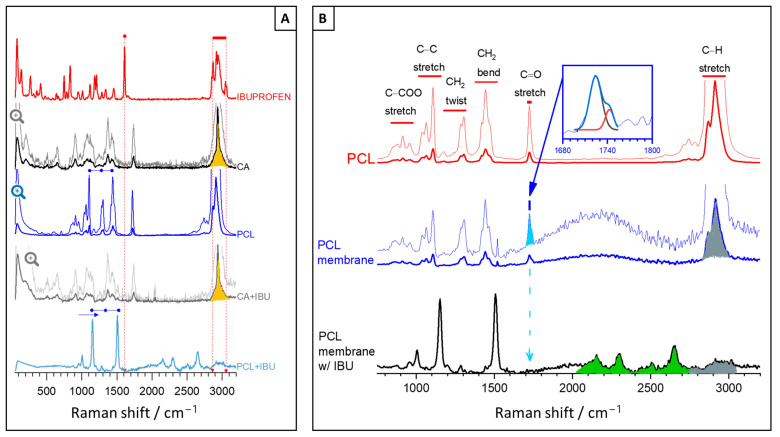
Raman spectra of (**A**) raw materials used to produce CA and PCL membranes and of membranes after ibuprofen release tests (CA + Ibu and PCL + Ibu); (**B**) PCL membrane and PCL membrane with Ibu compared to PCL powder. The spectra also show a magnification of the lowest-intensity peaks (lighter line).

The Raman spectra of Ibu, CA, and PCL raw materials are depicted in Figure 2A as well as the CA and PCL membranes’ spectra after Ibu release. In these membranes, the characteristic peaks of Ibu (e.g., at 1607 cm^−1^ and 2865–3060 cm^−1^ for the C=C stretching, and methyl groups C–H stretch + carboxyl group OH stretch, respectively) are absent, suggesting a complete drug release or retention at trace levels. Other Ibu characteristic peaks (magnified in the lighter line), primarily attributed to aryl ring stretching, are present at 413 cm^−1^, 636 cm^−1^, 748 cm^−1^, 833 cm^−1^, 948 cm^−1^, 1010 cm^−1^, 1184 cm^−1^, 1211 cm^−1^, and 1576 cm^−1^. The Ibu peaks of medium intensity at 266 cm^−1^, 662 cm^−1^, 783 cm^−1^, 820 cm^−1^, 943 cm^−1^, 1116 cm^−1^, 1341 cm^−1^, and 1452 cm^−1^ are due to bending, twisting, or rocking of C_x_–H_y_ [18]. 

The spectra in Figure 2A primarily exhibit peaks corresponding to the polymer vibration modes as detailed in Table S1. While many of these modes are common to both polymers, the peaks associated with CH, CH_2_, and CH_3_ stretching modes exhibit markedly different shapes. In CA, the peaks exhibit a typical triangular-like shape at the base, marked in yellow as a characteristic peak that readily confirms its presence in the membrane. In contrast, the PCL membrane multipeak is prominent only prior to loading with the Ibu drug. This is shown in Figure 2B, along with an apparent loss of the peak corresponding to the C=O band (marked in blue) and the emergence of new peaks (marked in green), possibly due to the formation of new chemical species. This suggests that the resulting material may no longer be considered PCL and may be more amorphous, as the multipeak at higher frequencies almost vanished (marked in grey), and it is often referred to as the PCL crystalline fraction [23].

In comparison, the CA membranes before and after Ibu loading exhibit remarkable similarity, with no discernible differences noted. Consequently, only the results after Ibu release are presented. Despite being immersed in water, the CA membrane displays notable stability, maintaining a composition akin to the raw powder material, even when considering peak widths and deducing information regarding changes in crystallinity. Conversely, the same conclusion cannot be drawn for PCL. Notably, the prominent bands, such as the C–C stretching (1110 cm^−1^), CH_2_ twisting (1299 cm^−1^), and CH_2_-bending modes (1441 cm^−1^), shift to the right, indicating that interactions between the functional groups of PCL and Ibu, such as van der Waals or hydrogen bonding, may have occurred [24]. Additionally, the peak assigned to the strong carbonyl stretching mode (C=O) apparently vanished, and new chemical species or complexes seems to have formed in low concentrations. These phenomena are highlighted in Figure 2B and may indicate chemical interaction of PCL with Ibu or even PCL hydrolysis (degradation), potentially leading to the formation of carboxylic acid groups, typically exhibiting peaks in the range of 2154–2300 cm^−1^ or both. Although unequivocal identification was not feasible, the absence of C=O groups and significant intensity reduction of the highest-frequency peak suggest significant chemical modification and degradation of the polymer, potentially resulting in increased hydrophilicity and a more amorphous structure. Both hydrophilicity and amorphous structure are advantageous characteristics for drug delivery applications, as they improve drug solubility, facilitate faster release kinetics, and potentially offer better control over drug release profiles.

Nonetheless, prior to drug release, the PCL membrane retained the main crystallinity characteristics of the raw material, similarly to CA. This was further confirmed by determining the crystallinity of the unloaded PCL membrane through spectral analysis, specifically through the deconvolution of the C=O stretching band in the region 1710–1750 cm^−1^ into two Gaussian lines centred at 1724 cm^−1^ and 1741 cm^−1^ as shown in the inset of Figure 2B. The first peak corresponds to the crystalline phase, whereas the latter is assigned to amorphous domains. The fraction of the crystalline phase $X_{c}$ was calculated according to the equation: $X_{C}=I_{C}/\left(I_{C}+I_{A}\right),$ where $I_{C}$ and $I_{A}$ refer to the integrated intensity of crystalline and amorphous components [23]. Hence, before Ibu loading, the crystalline phase $X_{c}$ of the PCL membrane was approximately ~70%. This suggests that the drug release rate would be slower than that provided by a more amorphous material of similar molecular weight. However, since the composition and structure of the PCL membrane changed after immersion in water and subsequent drug release to an undetectable level, it can be concluded that the interaction was beneficial and aligns with the subsequent release profiles presented.

**Figure 3:**
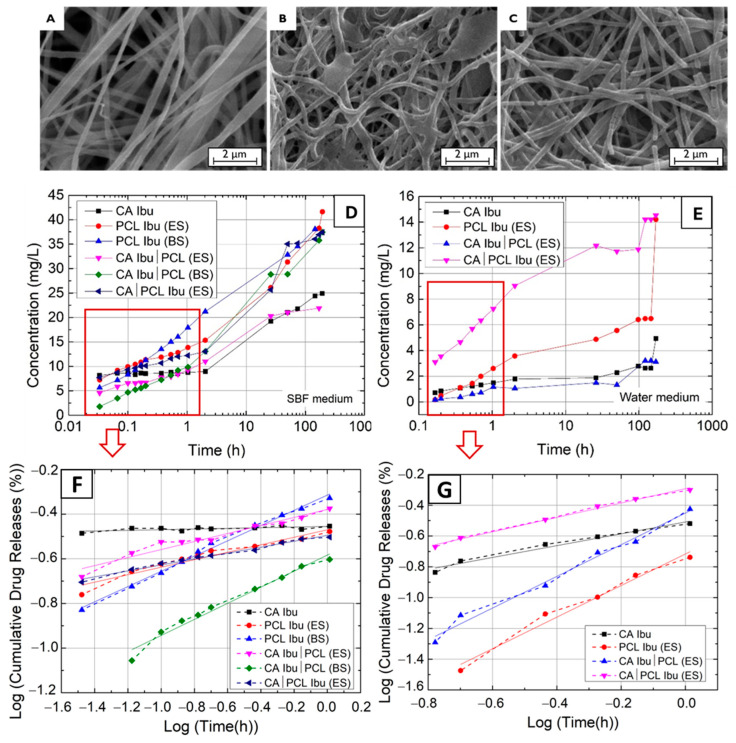
SEM images of samples following 190 h of drug release: (**A**) CA Ibu; (**B**) PCL Ibu; (**C**) CA|PCL Ibu. Drug release of various membranes versus time: (**D**) in SBF medium; (**E**) in water medium. Fitting experimental data following 1 h of drug release to the Korsmeyer–Peppas model: (**F**) in SBF medium; (**G**) in water medium.

The drug release profile with time of the different membranes is shown in Figure 3. The release tests were performed over 190 h in two media: SBF (Figure 3D), which mimics body fluids and water (Figure 3F) as a reference non-ionic medium. As the release mechanisms are defined in the first hour of release, Figure 3E,G show the release plot (points and dashed line) within this period and the corresponding fitting line (full line). In addition, the SEM images show the morphology of the membranes after 190 h of release for CA, PCL, and CA|PCL.

A slower drug release from the CA membranes was observed and CA Ibu membranes covered with PCL membranes showed a lower quantity of drug release at the beginning, but thereafter they followed a similar trend. This means that, initially, the PCL membrane may absorb the Ibu released from the membrane beneath it, but then it performs like a PCL + Ibu (ES) membrane. Comparing the behaviour of PCL membranes made with ES and BS, the PCL (BS) membrane shows a linear release over time, whereas the PCL (ES) membrane shows low variation up to 2 h, after which the release is also faster. Due to porosity, PCL dissolution is faster in BS membranes, whereas, in ES, it takes more time to start dissolving PCL and then release the drug. Therefore, the BS membrane, due to its greater porosity, allows faster drug release at the beginning and releases a high concentration at the end. This seems to be in line with the Raman results as these suggest PCL degradation after drug release, becoming more amorphous. Comparing the release behaviour of PCL ES and BS deposited on top of a CA Ibu membrane, the results show a slower drug release at the initial release time, but, after two 2 h, the samples CA|PCL (ES) release a lower concentration than CA|PCL (BS), which is consistent with the higher porosity of the BS membranes and in the two media SBF and water. Also, if we compare the SEM images of the respective membranes before and after the release, they reveal that, after the release, the fibres were reduced in size and several had broken. In addition, it has been verified whether the release of PCL Ibu on the top of a CA membrane will lead to adsorption of the drug by the CA membrane; it may not, because the drug release profile of the two layers of membranes is similar to that of the PCL membrane alone.

Considering the volume of media employed in the release experiments, the overall quantity of drug released was calculated, and the findings are presented in Table 1. Notably, the Ibu release over the specific period was greater in PCL Ibu membranes for both media (SBF and water). Furthermore, PCL on the CA Ibu membranes decreased the initial total release. This suggests that a stacked configuration of bilayers, CA + Ibu|PCL, could improve temporal control of the drug release and prevent biofilm formation, making it a compelling avenue for further investigation.

On the other hand, upon examining the drug release models aligned with the curves in Figure 3, it was confirmed that the most suitable fit for Figure 3E,G in the initial 60 min was the Korsmeyer–Peppas $M=Kt^{n}$, where M is the quantity of drug released over time t, K is a constant related with structural modifications and geometric characteristics of the system (also perceived as the release velocity constant), and n determines the drug release mechanism [25]. Should n be less than 0.5, under the quasi-Fickian model, the release mechanism is dominated by diffusion, occurring partly within the swollen matrix and partly in the solvent-filled pores. Where n equals 0.5, in the Fickian model, the drug release mechanisms are primarily driven by diffusion, significantly surpassing the relaxation of the polymer chain. In scenarios where 0.5 < n < 1, under the non-Fickian model, we have anomalous transport: the drug release is governed equally by diffusion and swelling. Herein, the amalgamation of gradual polymer chain reorganisation and concurrent diffusion culminates in anomalous effects. For n exceeding 1, in the non-Fickian model, we observe a super case: a swift escalation in the swelling ratio is observed, stemming from the expansion of forces exerted by the swollen matrix [26]. Considering the values obtained for n, the release in SBF is predominantly diffusion-driven, but, in water, swelling plays a dominating role, whilst, in the case of PCL + Ibu in water, diffusion and swelling govern at similar rates.

**Figure 4:**
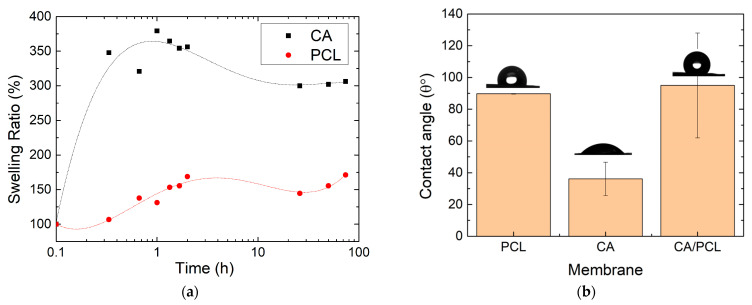
(**a**) Average percentage change in the mass of CA and PCL membranes in the swelling process when submerged in SBF (membranes without Ibu); (**b**) contact angle of PCL, CA, and CA/PCL (membranes produced by ES without Ibu).

The drug release is related to swelling of membranes when immersed in SBF solutions. The average percentage change in the mass of the CA and PCL membranes in the swelling process when submerged in SBF is shown in Figure 4a. The swelling of the CA membrane showed a rapid increase, reaching a maximum of 379% after 1 h. The swelling decreased with increasing swelling time, likely related to the drying method and possible loss of mass of the membranes during drying with paper. The PCL membranes showed a lower swelling ratio throughout the process, reaching a maximum of around 150% after 2 h. The low swelling ratio is expected because PCL is reported to be hydrophobic, meaning it has low wettability; however, in this case, water is captured from the pores of membrane. Since the measurements were all performed at 37 °C, polymer degradation is not influencing the swelling or the drug release. PCL degradation is around 58 °C [27], while for CA it is above 300 °C [28].

From contact angle measurements, shown in Figure 4b, the CA membrane is hydrophilic, with water spreading immediately upon contact (θ = 36.13 ± 10.5°). The PCL membrane exhibited low wettability (θ = 89.75 ± 0.2°); however, after 5–6 min, the drop spread, and the membranes became wet, as shown in Figure S9 in the Supporting Information. The wettability of the CA and PCL double-layer membrane was measured on the PCL side, and, as anticipated, it initially displays hydrophobicity (θ = 95.02 ± 33°).

Furthermore, in terms of drug release, coatings applied to metallic implants must demonstrate sturdy adhesion qualities to guarantee enduring durability, both throughout the manufacturing phase and subsequent implantation. Consequently, mechanical tests were executed on both the standalone membranes and the SS substrates to which these membranes were applied. Tensile tests conducted on the individual membranes revealed varied mechanical behaviours. Notably, the CA membranes displayed brittle fracture characteristics, as indicated by minimal plastic deformation followed by a rapid stress drop. Conversely, the PCL membranes exhibited elastic deformation. While PCL is inherently more elastic than CA, its brittleness tends to increase when produced by the BS technique due to porosity. Table 2 lists the relevant results obtained for the membranes tested, including the average thickness, yield strength, and ultimate strength.

The PCL membranes produced via the BS method showed exceptional elasticity and strength. PCL is renowned for its high mechanical strength, while CA, by comparison, exhibits relatively inferior mechanical properties. As a results, the stacked layers of PCL on CA (mentioned as CAǀPCL) compensate for the inherent mechanical weaknesses of CA and achieve a harmonious balance between flexibility and hardness. In accordance with the existing literature [29], this enhancement is further bolstered by PCL’s sturdy mechanical characteristics, blend compatibility and uniformity, morphological improvements, heightened ductility, and the capacity to customise mechanical properties to meet specific application requirements.

To assess the membrane’s adhesion to the SS substrate surface, the substrates were securely fastened within a uniaxial tensile machine (see Supplementary Information). One end of each substrate was clamped, and a strip of tape was placed over the membrane, also secured by a clamp. All membranes were fabricated through the electrospinning process onto the substrates, with some including an additional electrosprayed layer of chitosan (CHI) between the substrate and the membrane to explore its impact on adhesion [16]. The detailed results are presented in Table 3.

When results are compared, it becomes evident that the CHI’s presence significantly improves membrane adherence to the substrate, with a more pronounced effect observed in the CA|PCL bilayer system. This observation is in line with previous research [15], which also recognised the marked enhancement in membrane adhesion following the introduction of CHI. The increased adhesion noted in this study is partly attributed to electrostatic interactions, enabled by the inherent charge polarity of the materials involved. Specifically, CA carries a negative charge, whereas the CHI layer is positively charged, thus promoting attractive electrostatic forces at the interface [30]. Following the peel-off tests, it was possible to qualitatively deduce that the substrates with CHI retained more membrane fragments, as depicted in the accompanying photographs in Table 3, while substrates without CHI showed minimal membrane remnants.

## 4. Conclusions

Cellulose acetate and polycaprolactone were chosen as the materials for testing drug retention due to their biocompatibility. The CA membranes were produced using electrospinning, both with and without the incorporation of 1 mg of Ibu. In contrast, the PCL membranes were fabricated using two different production techniques, electrospinning and blow spinning, with solutions either with or without 1 mg of Ibu. Raman spectroscopy was employed to analyse the composition of the CA and PCL membranes prior to Ibu loading and after Ibu release in water. The assessments of drug release were carried out using membranes applied onto stainless-steel substrates. To enhance membrane–substrate adhesion, the SS substrates underwent thorough cleaning and mechanical treatment. The membrane segments containing Ibu were immersed in either SBF or water, and spectral analyses of the SBF or water medium were conducted at regular intervals over a 7-day period.

A notable difference in drug release profiles was observed between PCL Ibu (ES) and PCL Ibu (BS), as well as between the bilayers CA Ibu|PCL (ES) and CA Ibu|PCL (BS). Specifically, the porosity of BS membranes led to accelerated dissolution and drug release for PCL, in contrast to the more gradual release seen in membranes prepared via ES. It was also clear that the PCL + Ibu membranes allowed for easier drug release compared to the CA + Ibu membranes.

PCL (BS) showed better elasticity and strength, while CA showed relatively poor mechanical properties. PCL deposited on CA resulted in an increase in tensile strength compared to using CA alone. This combination overcomes the poor mechanical properties of CA and achieves a reasonable balance between flexibility and hardness.

Adhesion tests showed that the inclusion of the CHI layer before CA and PCL deposition substantially increased the peel force, resulting in a range of 0.15–0.22 N for membranes without CHI and 0.21–0.74 N for membranes with CHI.

Overall, this study suggests that coatings containing a drug can be easily applied to metallic implants and engineered to control drug release in terms of methodology (ES or BS) and composition (with or without drug) and type of biocompatible polymer. Additional research is needed to confirm the effectiveness of various drugs and polymer layers in inhibiting biofilm formation.

## Figures and Tables

**Table 1:** Estimated drug released in SBF or water for each membrane studied and exponent factor (*n*) obtained from fitting the experimental data with a Korsmeyer–Peppas model equation.

	In SBF			In Water		
Membranes	Ibu Released (mg)	n	log(M) = n × log(t) + log(K)	Ibu Released (mg)	n	log(M) = n × log(t) + log(K)
CA Ibu	0.498	0.014	y = 0.014x − 0.455	0.098	0.384	y = 0.384x − 0.509
PCL Ibu (ES)	0.832	0.168	y = 0.168x − 0.470	0.284	1.026	y = 1.026x − 0.717
PCL Ibu (BS)	0.762	0.341	y = 0.341x − 0.315	-	-	-
CA Ibu|PCL (ES)	0.438	0.180	y = 0.180x − 0.379	0.062	1.029	y = 1.029x − 0.451
CA Ibu|PCL (BS)	0.789	0.360	y = 0.3601x − 0.583	-	-	-
CA|PCL Ibu (ES)	0.779	0.133	y = 0.133x − 0.495	0.290	0.468	y = 0.468x − 0.292

**Table 2:** Results of tensile tests performed on the various membranes.

Membrane	Thickness (μm)	Yield Strength (kPa)	Ultimate Strength (kPa)
CA	39 ± 15	-	0.86 ± 0.08
CA Ibu	53 ± 4	-	5.42 ± 0.74
PCL (ES)	27 ± 7	-	2.40 ± 0.11
PCL Ibu (ES)	67 ± 10	-	3.86 ± 0.18
PCL (BS)	89 ± 21	21.94 ± 0.64	89 ± 21
PCL Ibu (BS)	113 ± 23	13.83 ± 1.28	113 ± 23
CA Ibu|PCL (ES)	19 ± 2	-	1.84 ± 0.42
CA Ibu|PCL (BS)	45 ± 6	5.21 ± 0.45	45 ± 6

**Table 3:** Results of adhesion tests performed on the various membranes deposited on SS substrates and photographs of some samples after peel tests (A) with CHI and (B) without CHI layer.

Membranes on SS Substrates	Average Force		SS Substrates after Adhesion Test
	Without CHI	With CHI	
CA	0.15 ± 0.02	0.21 ± 0.03	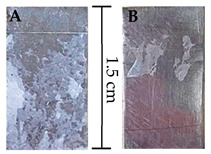
CA Ibu	0.20 ± 0.03	0.22 ± 0.03	
PCL	0.20 ± 0.03	0.30 ± 0.02	
PCL Ibu	0.22 ± 0.02	0.27 ± 0.03	
CA|PCL	0.21 ± 0.04	0.46 ± 0.06	
CA Ibu|PCL Ibu	0.22 ± 0.05	0.74 ± 0.05	

## Data Availability

The raw data supporting the conclusions of this article will be made available by the authors on request.

## Supplementary Materials

The following supporting information can be downloaded at https://www.mdpi.com/article/10.3390/polym16142006/s1. Figure S1: Optical microscope image of stainless-steel substrate: (a) Before treatment and (b) after treatment. Figure S2: Experimental setup of electrospinning/electrospray: (1) collector with four SS substrates (2) attached to it, (3) copper ring, and (4) syringe needle. Figure S3: CA membrane with Ibu produced by electrospinning. (a) Photograph of the membrane; (b) optical microscope image of the fibres. Figure S4: Representation of the studied samples: single and multilayers with and without Ibu deposited in SS substrates, and the drug release tests performed. Table S1: Identification of prominent peaks in the Raman spectra of electrospun cellulose acetate (CA) and polycaprolactone (PCL) membranes, according to references. Figure S5: (a) Membranes of CA Ibu as electrosprayed on SS substrates, (b) PCL Ibu before release, (c) PCL Ibu after release. Figure S6: Absorption spectra for the different ibuprofen concentrations on SBF and (b) calibration curve of the two obtained peaks on SBF. Figure S7: (a) Image of the tensile test and (b) an example curve obtained for PCL (BS). Figure S8: (a) Schematics of the peeling-off tests and (b) an example of curve obtained for CHI|CA Ibu|PCL Ibu. Figure S9: Variation of contact angle with time of PCL (ES) membrane.
