# Supplementary material for: Cellulose Acetate and Polycaprolactone Fibre Coatings on Medical-Grade Metal Substrates for Controlled Drug Release
Source: Polymers (Basel). 2024 Jul 13;16(14):2006. doi: 10.3390/polym16142006 (PMC11280613; doi:10.3390/polym16142006)
Supplement: Supplementary file 1 [file polymers-16-02006-s001.zip › polymers-3069075-supplementary.pdf]

Supporting Information

Experimental Details

S1. Stainless-Steel Substrates

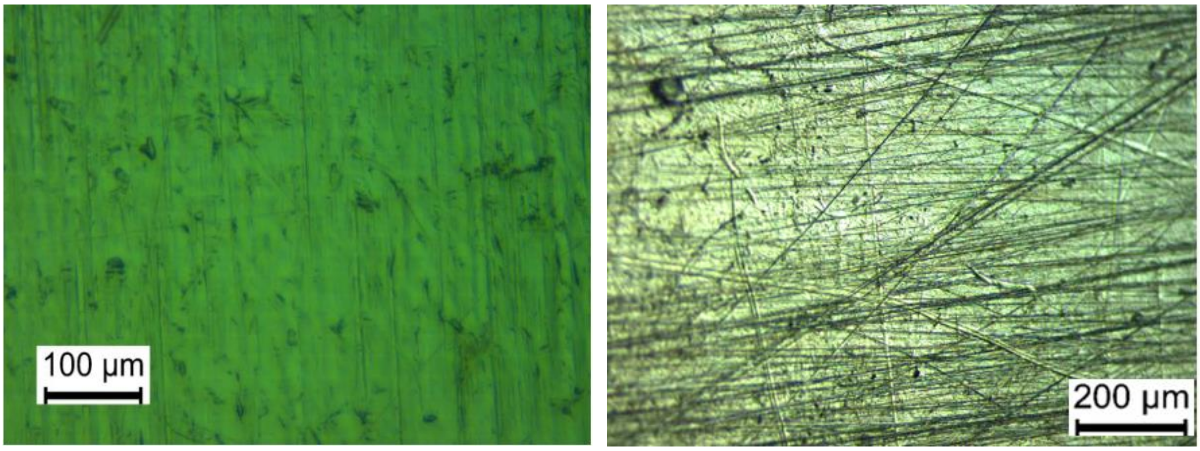

(**a**) (**b**)

Figure S1. Optical microscope image of stainless-steel substrate: (a) before treatment and, (b) after treatment [1]

S2. Membranes Preparation

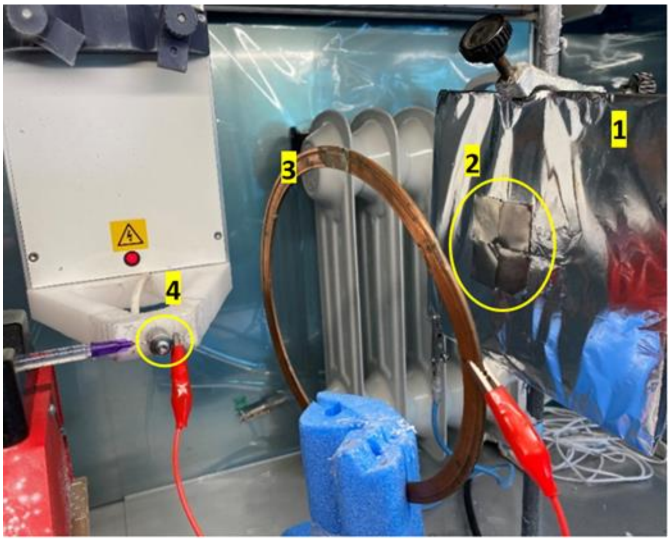

Figure S2. Experimental setup of electrospinning/electrospray: (1) collector with four SS substrates (2) attached to it; (3) copper ring and (4) syringe needle.

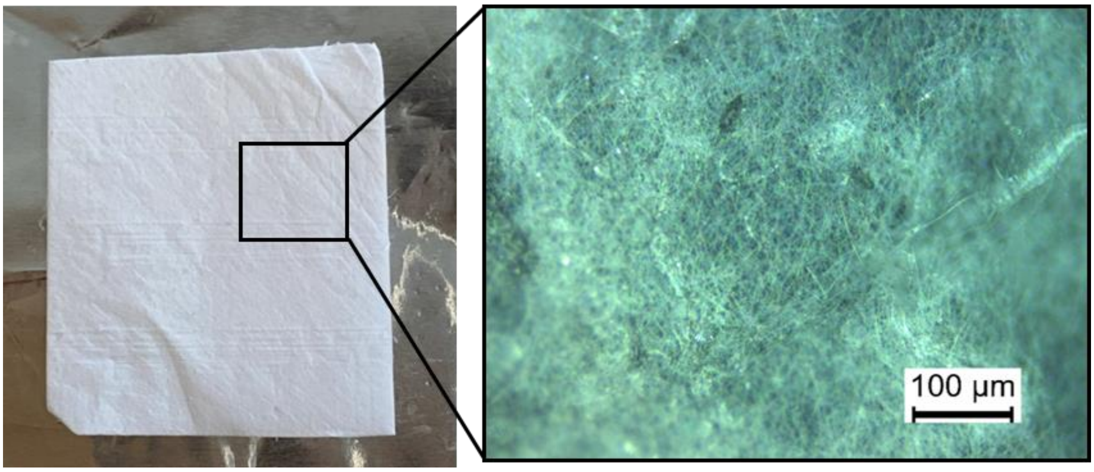

(**a**) (**b**)

Figure S3. CA membrane with Ibu produced by electrospinning. (a) Photograph of the membrane; (b) Optical microscope image of the fibres.

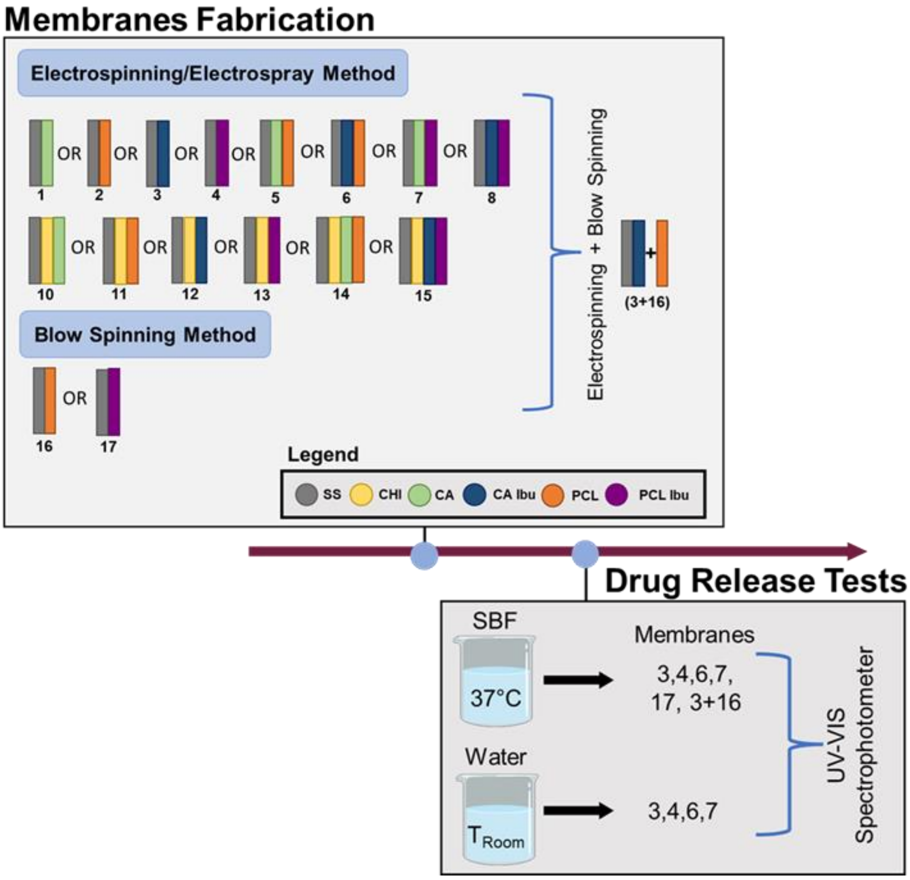

Figure S4. Representation of the studied samples: single and multilayers with and without Ibu deposited in SS Substrates, and the drug release tests performed.

S3. Morphological and chemical characterization

Table S1. Identification of prominent peaks in the Raman spectra of electrospun cellulose acetate (CA) and polycaprolactone (PCL) membranes, according to references [2,3]

Vibration Modes			
**CA**		**PCL**	
**Peak Position (cm^{-1})**	**Assignment**	**Peak Position (cm^{-1})**	**Assignment**
651-384	skeletal C–O–C, C–C–C, O–C–C and O–C–O bonds	-	-
-	-	865	Amorphous phase
904	C–O–C in plane, symmetric	909, 963	C–COO skeletal stretching modes
1073	C–C and C–O stretch	1037, 1064, 1110	C–C stretching modes
1251	H–O–C	1299, 1304	Doublet peak twisting of CH_2 group derived from crystalline and amorphous PCL planes respectively
1372	H–C–C, H–C–O	-	-
1425	H–C–H and H–O–C bonds	1418, 1441	CH_2 bending vibration specific to methylene linkages
1738	C=O stretching band	1721	C=O stretching band
2937	CH, CH_2 stretch	2800-3200	CH, CH_2 and CH_3 stretching modes

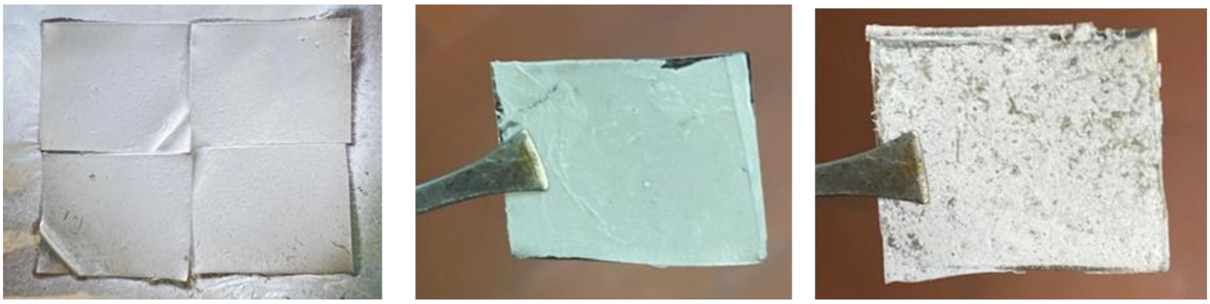

(**a**) (**b**) **(c)**

Figure S5. (a) Membranes of CA Ibu as electro-sprayed on SS substrates, (b) PCL Ibu before release, (c) PCL Ibu after release.

S4. Drug Release Methodology

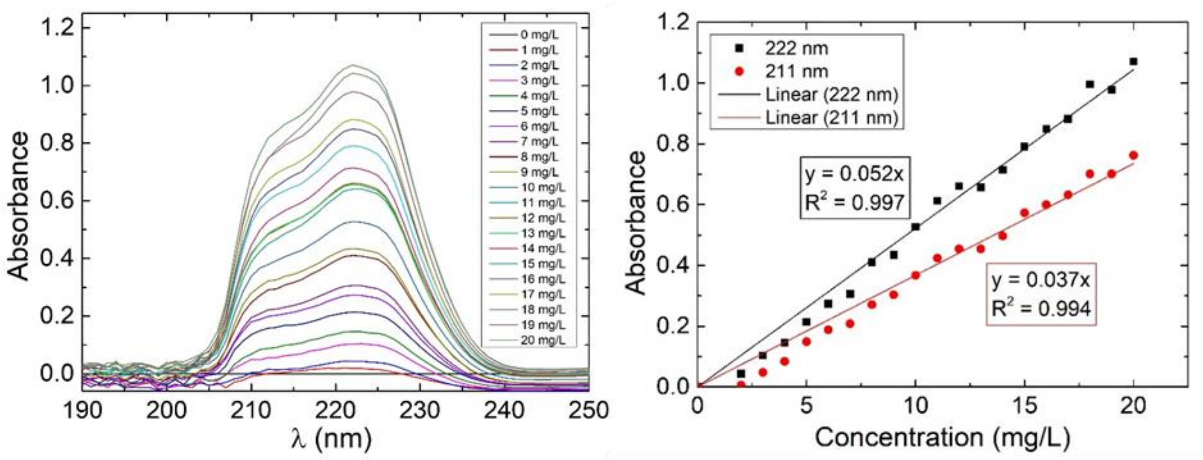

(**a**) (**b**)

Figure S6. (a) Absorption spectra for the different ibuprofen concentrations on SBF and (b) calibration curve of the two obtained peaks on SBF.

The average variation of the drug release concentration over time across the different membranes immersed in SBF or water was determined using equations S1 and S2 resulting from the linear fitting of the calibration curves [3]. To estimate the amount of Ibu released, the final concentration values obtained for each membrane were multiplied by 20 mL, the volume of the medium in which each membrane was immersed.

$$\text{Concentration of IBU in SBF for 211 nm peak (mg/L)} = \frac{\text{Absorbance}}{0.037} \tag{S1}$$

$$\text{Concentration of IBU in water for 211 nm peak (mg/L)} = \frac{\text{Absorbance}}{0.0378} \tag{S2}$$

The experimental data was fitted to the Korsmeyer-Peppas model equation, plotting the fraction of drug released (M) against time (t) in a logarithmic scale, to obtain the release kinetics and understand the drug release mechanism. The fractions of drug released were calculated by dividing the amount of drug released at various time points (M_t) by the total amount of drug released (M_∞), following the equation S3.

$$\mathrm{M} = \frac{M_t}{M_\infty} = \frac{\text{Concentrarion at each time}*\text{20mL}}{M_\infty} \tag{S3}$$

S5. Mechanical Tests

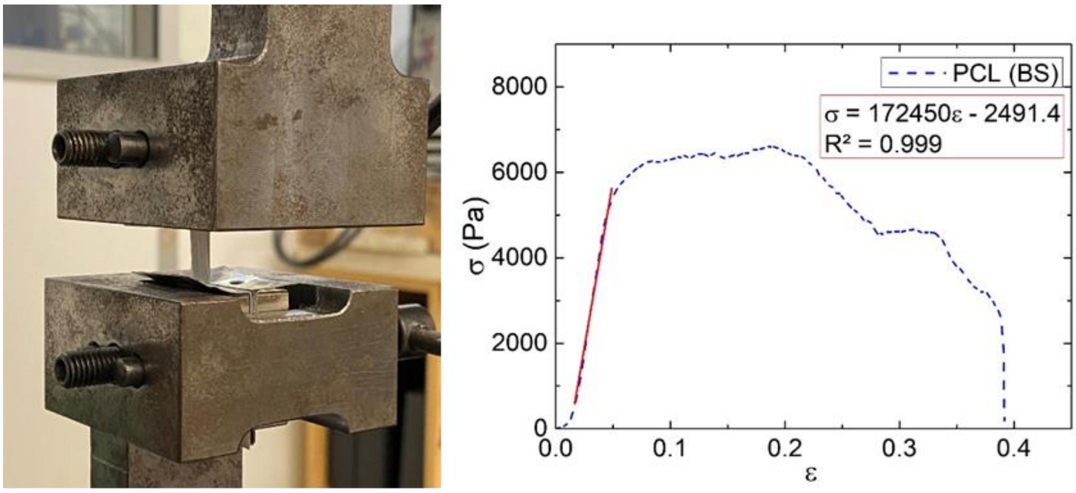

(**a**) (**b**)

Figure S7. (a) Image of the tensile test and (b) an example curve obtained for PCL (BS).

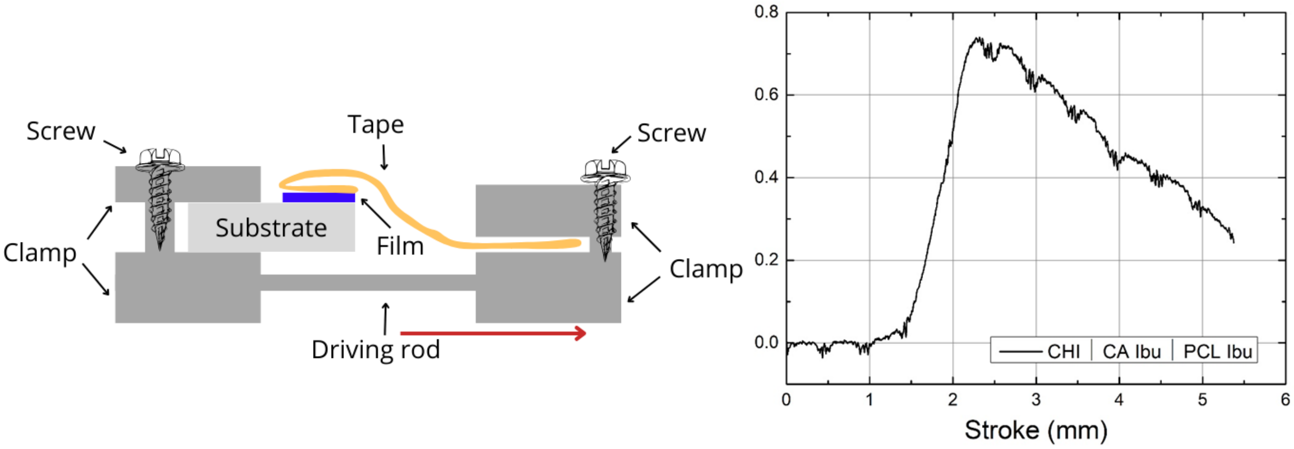

(**a**) (**b**)

Figure S8. (a) Schematics of the peeling-off tests and (b) an example of curve obtained for CHI | CA Ibu | PCL Ibu

S6. Contact Angle Measurements

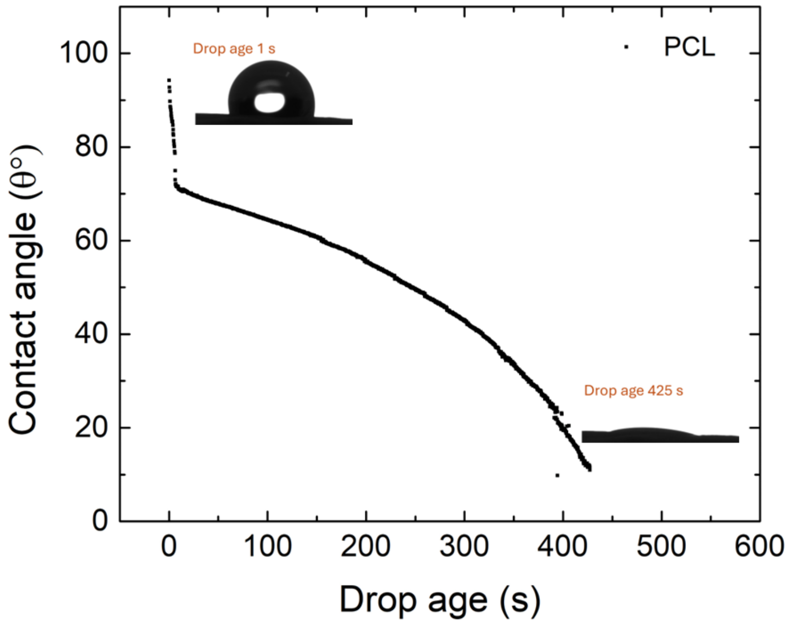

Figure S9. Variation of contact angle with time of PCL (ES) membrane.

References

1. Faria, J.; Dionísio, B.; Soares, Í.; Baptista, A.C.; Marques, A.; Gonçalves, L.; Bettencourt, A.; Baleizão, C.; Ferreira, I. Cellulose Acetate Fibres Loaded with Daptomycin for Metal Implant Coatings. *Carbohydr Polym* **2022**, *276*, doi:10.1016/j.carbpol.2021.118733.

2. Baranowska-Korczyc, A.; Warowicka, A.; Jasiurkowska-Delaporte, M.; Grześkowiak, B.; Jarek, M.; Maciejewska, B.M.; Jurga-Stopa, J.; Jurga, S. Antimicrobial Electrospun Poly(ε-Caprolactone) Scaffolds for Gingival Fibroblast Growth. *RSC Adv* **2016**, *6*, 19647–19656, doi:10.1039/c6ra02486f.

3. Baptista, A.C.; Brito, M.; Marques, A.; Ferreira, I. Electronic Control of Drug Release from Gauze or Cellulose Acetate Fibres for Dermal Applications. *J Mater Chem B* **2021**, *9*, 3515–3522, doi:10.1039/d1tb00249j.
